# P224: The influence of maximal barrier precaution on the prevention of central line-associated bloodstream infection in intensive care units

**DOI:** 10.1186/2047-2994-2-S1-P224

**Published:** 2013-06-20

**Authors:** J Moon, S Kim, J Im, J Baek, J Lee

**Affiliations:** 1 Department of Infection Control, Inha University Hospital, Korea, Republic Of; 2 Division of Infectious Disease, Inha University, Incheon, Korea, Republic Of

## Introduction

Although maximal barrier precaution (MBP) is recommended for the prevention of central line-associated bloodstream infection (CLABSI), use of MBP for the insertion of central venous catheter (CVC) in our hospital was very low.

## Objectives

This study was intended to see whether improving the compliance for MBP during CVC insertion could reduce the incidence of CLABSI in the intensive care units (ICU).

## Methods

This study was conducted at 5 intensive care units (total 53 beds, 2 medical ICUs, 1 cardiac ICU, 2 surgical ICUs) in one teaching hospital from Mar 2012 to Nov 2012. Infection control team educated proper practice on MBP during CVC insertion, monitored the CVC insertion procedure by using electronic check-list system, and provided feedback to physicians. The CDC-National Healthcare Safety Network definition for CLABSI was used. Chi-square test was done using Epi-info 6.0(Centers for Disease Control and Prevention, Atlanta, GA).

## Results

MBP compliance rate during CVC insertion was 38.8% before implementing this intervention. A total of 440 CVCs were undertaken for 9 months. Compliance rate was increased from average 47.8% of the first quarter(Mar~May, 2012) to 79.6% of the third quarter(Sep~Nov, 2012) (*p*<*0.001*). The incidence of CLABSI was significantly improved from 4.00 (11/2,748 catheter days) per 1,000 catheter days during the first quarter to 0.70(2/2,817 catheter days) per 1,000 catheter days(*p*=*0.011*) at the third quarter.

## Conclusion

MBP compliance for CVC insertion has increased through education, monitoring and feedback strategies with achieving the reduction of CLABSI.

## Disclosure of interest

None declared

